# Long‐term anxiety and depression signatures of participants that received esophageal cancer screening: A multicenter population‐based cohort study

**DOI:** 10.1002/cam4.5404

**Published:** 2022-11-24

**Authors:** Juan Zhu, Shanrui Ma, Ru Chen, Shuanghua Xie, Zhaorui Liu, Zhengkui Liu, Wenqiang Wei

**Affiliations:** ^1^ National Central Cancer Registry, National Cancer Center/National Clinical Research Center for Cancer / Cancer Hospital Chinese Academy of Medical Sciences and Peking Union Medical College Beijing China; ^2^ Department of Cancer Prevention The Cancer Hospital of the University of Chinese Academy of Sciences (Zhejiang Cancer Hospital), Institute of Cancer and Basic Medicine (IBMC), Chinese Academy of Sciences Hangzhou China; ^3^ Key Laboratory of Mental Health, Ministry of Health (Peking University) Institute of Mental Health, Peking University Beijing China; ^4^ Key Laboratory of Mental Health Institute of Psychology, Chinese Academy of Sciences Beijing China

**Keywords:** anxiety, cohort, depression, endoscopic screening, esophageal cancer, high‐grade intraepithelial neoplasia, high‐incidence areas, long‐term, psychological distress

## Abstract

**Background:**

Current evidence on the psychological impact of screening and diagnosis of esophageal cancer (EC) is limited and unclear.

**Methods:**

This multicenter, population‐based, prospective study was conducted in five high‐incidence regions in China from 2017 to 2020. The screened participants were diagnosed as healthy, esophagitis, low‐grade intraepithelial neoplasia (LGIN), high‐grade intraepithelial neoplasia (HGIN), or EC based on pathological biopsy. The psychological impact of the screening was assessed by comparing anxiety and depression symptoms at baseline and follow‐up.

**Results:**

A total of 1973 individuals were ultimately included, with an average follow‐up of 22.2 months. The prevalence of anxiety and depression symptoms in screened population at baseline was 14.3% and 18.4%. The prevalence of anxiety and depression symptoms of screeners at follow‐up declined (all *p* < 0.001). The anxiety (RR [95% CI]: 0.37 [0.30–0.46]) and depression (0.29 [0.24–0.36]) of screeners weakened over time, but the anxiety and depression symptoms was continuous for patients with HGIN and patients with EC. Compared with the participants classified as normal, the RRs(95% CI) of anxiety and depression symptoms were 2.20 (1.10–4.30) and 2.03 (1.07–3.86) for the patients with HGIN and 2.30 (0.82–6.20) and 3.79 (01.71–8.43) for the patients with EC.

**Conclusion:**

The anxiety and depression symptoms of screeners weakened over time, except in patients with HGIN and EC, for whom it remained lasting and high. Psychological assistance and interventions are urgently needed for individuals who are ready for screening and for those diagnosed as having HGIN or EC.

## 1 INTRODUCTION

Esophageal cancer (EC) is a common type of upper gastrointestinal cancer that contributes to the worldwide cancer burden, especially in developing countries.^1^ According to the GLOBOCAN report, there were an estimated 572,034 new EC cases and 508,585 deaths related to EC in 2018,^2^ with approximately half of all the new cases occurring in China.^2^ The overall 5‐year survival for patients with EC is low in China, at only 30.3%.^3^ The survival of EC patients is closely related to the stage at diagnosis.^4^ Strong evidence from large population‐based studies has confirmed the effectiveness of endoscopic screening in reducing EC incidence and mortality.^5^, ^6^ Japan and South Korea have carried out nationwide upper gastrointestinal screening programs,^7^, ^8^ while endoscopic screening is only launched in high‐incidence regions (those with a higher risk for EC) in China.^5^, ^6^

Although endoscopic screening of high‐incidence groups has been proven to be beneficial in lowering mortality and improving survival in EC, accompanying negative psychosocial consequences of screening (i.e., anxiety and depression symptoms) may raise questions about the overall benefit of endoscopic screening.^9^ To date, the psychosocial consequences of cancer screening have been largely underestimated.^10^ The negative impact of endoscopic screening is mainly reflected in two aspects. First, the invasive endoscopy is a stressor, increasing screeners' anxiety.^11^ Second, screened patients worry about their screening and diagnosis results.^12^, ^13^ However, thus far, there is limited and insufficient reliable evidence on long‐term anxiety and depression signatures of endoscopic screeners. To optimize EC screening and minimize potential harm, psychological consequences should always be considered when a mass screening is introduced. Therefore, we carried out a population‐based, multicenter, prospective study to evaluate the long‐term anxiety and depression symptoms of endoscopic screening and diagnosis.

## 2 METHODS

### 2.1 Study design and participants

**FIGURE 1:**
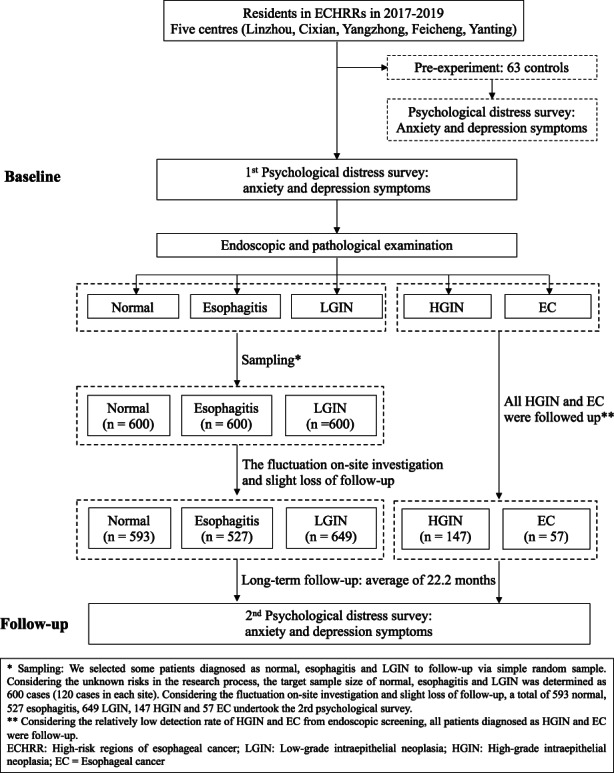
Flowchart of EC cohort

We carried out a multicenter follow‐up study in 5 areas of China where the risk of EC is high (Linzhou, Cixian, Yangzhong, Feicheng, and Yanting). The study flowchart is shown in Figure 1. The inclusion criteria were as follows: (1) Community residents aged 40–69 years old; (2) voluntary participation in the study with signed informed consent; (3) an ability to understand the survey procedure; and (4) no serious vision or hearing problems. The exclusion criteria were as follows: (1) a diagnosis of EC or other cancers and (2) contraindications to endoscopy, such as acute perforation of the upper digestive tract, severe heart, lung, kidney, or brain dysfunction, or multi‐organ failure.

1. At baseline (between May 2017 and October 2019), permanent residents aged 40–69 years were recruited to have their anxiety and depression symptoms evaluated and undergo endoscopic screening for EC. Those residents were invited by local well‐trained investigators. Those individuals who had no emergency symptoms and no history of cancer were included after signing informed consent forms. According to the endoscopy findings and pathological results, the eligible participants were classified as normal, esophagitis, LGIN, HGIN, or EC.

1. Follow‐up was performed from December 2019 to October 2020, with an average follow‐up of 22.2 months. (a) Considering the relatively high detection rate of normal or esophagitis or LGIN, we selected some patients classified as normal or diagnosed with esophagitis or LGIN for follow‐up via a simple random sample. The sample size was calculated according to the following formula. The minimum sample size of normal was calculated as 526. Considering the unknown risks in the research process, we determined the sample size of normal or esophagitis or LGIN was 600. Considering the total of five sites, the sample of normal or esophagitis or LGIN were distributed to each site evenly (120 cases/per site).

$$\frac{{z_{\alpha }}^{2}\times \mathrm{pq}}{d^{2}}$$

where *p* is the proportion of anxiety symptoms of normal, *p* = 5.21%,^14^ *q* = 1−*p*, *zα* = 1.96, power = 0.95, *d* = 0.04. (b) Considering the relatively low detection rate of HGIN and EC from endoscopic screening, all patients diagnosed with HGIN or EC were followed up.

### 2.2 Measurement

Prior to the endoscopic examination, a uniform questionnaire was used to collect information related to the participants' risk exposures at baseline (e.g., smoking, alcohol drinking, dietary habits) and their psychological status (anxiety and depression symptoms) at both baseline and follow‐up. Comparisons of anxiety and depression symptoms in the screening population at baseline and follow‐up were performed to assess the prevalence of anxiety and depression in different screening groups and to evaluate the psychological impact of cancer screening and diagnosis.

#### 2.2.1 Anxiety and depression symptoms

Anxiety symptoms were evaluated using the Chinese version of the seven‐item Generalized Anxiety Disorder (GAD‐7), a measurement tool widely used and acknowledged around the world. The GAD‐7 is used to identify anxiety symptoms of individuals in the past 2 weeks, with 7 items and 4 response scores (0–3). The prevalence of depression was assessed using the GAD‐7 scoring algorithm. A score of 5 was regarded as the threshold for positive anxiety symptoms.^15^ The nine‐item Patient Health Questionnaire (PHQ‐9), with 9 items and 4 response scores (0–3), corresponds to the Statistical Manual of Mental Disorders‐IV (DSM‐IV) diagnostic criteria for major depressive episodes. The response score ranges from 0 to 3. If the total score is higher than 5, it is considered as the presence of depression symptoms.^16^

The studies have shown the tool's good reliability and validity in primary medical care and clinical practice.^14^, ^16^ The GAD‐7 showed good reliability and validity in the target population of our study (Cronbach's alpha = 0.903, KMO = 0.901, Bartlett's test of sphericity *p* < 0.001). The PHQ‐9 showed good reliability and validity in the study (Cronbach's alpha = 0.907, KMO = 0.895, Bartlett's test of sphericity *p* < 0.001).

### 2.3 Quality control

The psychological outcomes of participants were evaluated by well‐trained investigators. We conducted a series of training programs for the investigators. Face‐to‐face or self‐administered interviews were conducted or managed by well‐trained local interviewers to ensure that the questionnaire was fully completed. The training was first conducted in a centralized way to ensure uniformity. Then the training team went to each center for further training possible if needed. Certification of the interviewers was carried out at the end of the training process. The interviewer completed a specific questionnaire according to the simulated on‐site survey and compared it with the standard answer. Certification could only be approved when the consistency of the survey is met. A total of 53 well‐trained investigators and interviewers, 72 qualified endoscopists and nurses, 27 qualified pathologists were equipped in the study. The screening outcome of participants was diagnosed by endoscopists and pathologists. Details on the key quality assurance and quality control procedures were shown in a previously published article.^17^

### 2.4 Statistical analysis

Considering the fluctuation of onsite investigations and the slight loss of participants at follow‐up, a total of 593 normal participants, 527 esophagitis patients, 649 LGIN patients, 147 HGIN patients, and 57 EC patients completed the second psychological survey. Absolute frequencies and percentages are presented for the categorical variables. Mean values (95% CIs) were used to describe the scores of anxiety and depression symptoms. Comparisons of the scores of anxiety and depression symptoms in the screened participants between baseline and follow‐up were made by the Wilcoxon matched‐pair signed‐ranks test. McNemar's test was performed to compare the prevalence of anxiety and depression symptoms of the participants at baseline and follow‐up. The anxiety and depression symptoms scores among groups with different grades of esophageal pathology were compared using the Kruskal–Wallis test. A chi‐squared test was run to test the prevalence of anxiety and depression symptoms among esophageal pathological grades.

Relative risk (RR) was calculated to estimate the long‐term impact of screening on anxiety and depression symptoms in the population over time. Cox regression was performed, and region, age, sex, household income, smoking, alcohol consumption, and self‐rated health were adjusted. Pairwise comparisons between the screening groups were conducted. Bonferroni's adjustment was used to compare the prevalence for each group. Data management, programming, and analyses were carried out using SAS 9.4 (SAS Institute Inc.). All tests of significance were two‐tailed. *p* < 0.05 was considered to indicate statistical significance. In addition, we evaluated the quality of the study on the basis of the Newcastle–Ottawa Scale (NOS) and the checklist of STROBE statements.

## 3 RESULTS

### 3.1 Participant characteristics

A total of 1973 eligible subjects with complete basic characteristics and twice psychological information were ultimately included (593 normal [30.1%], 527 esophagitis [26.7%], 649 LGIN [32.9%], 147 HGIN [7.5%], and 57 EC [2.9%]). The average follow‐up was 22.2 months (normal: 20.6 months, esophagitis: 23.5, LGIN: 22.2, HGIN: 23.4, EC: 24.2). The mean age was 59.4 years, and just over half of the participants were female (55.1%). The prevalence of anxiety symptoms in Cixian, Yangzhong, Feicheng, Linzhou, and Yanting was 14.2%, 3.2%, 4.0%, 46.0%, and 19.9% (*p* < 0.001), the corresponding prevalence of depression symptoms was 13.9%, 1.5%, 3.4%, 68.8%, and 36.8% (*p* < 0.001). Differences in anxiety and depression symptoms were found by sex, household income, smoking, alcohol consumption, and self‐rated health (all *p* < 0.05). The results are displayed in Table 1.

**TABLE 1:** Baseline characteristics of the screening participants

Variables	*N* (%)	Anxiety symptoms		Depression symptoms	
		Prevalence (%)	*p*	Prevalence (%)	*p*
Total sample	1973	14.3		18.4	
Age, years (Mean ± SD)	59.4 ± 7.5				
High risk regions					
East: Cixian	648 (32.8)	14.2	<0.001	13.9	<0.001
East: Yangzhong	537 (27.2)	3.2		1.5	
East: Feicheng	324 (16.4)	4.0		3.4	
Central: Linzhou	263 (13.3)	46.0		68.8	
West: Yanting	201 (10.2)	19.9		36.8	
Gender					
Male	886 (44.9)	11.5	0.001	16.4	0.030
Female	1086 (55.1)	16.7		20.2	
Marital status					
Married	1814 (91.9)	14.2	0.605	18.6	0.619
Other	159 (8.1)	15.7		17.0	
Highest education level					
Primary school or below	1043 (52.9)	14.7	0.659	20.0	0.140
Junior or Senior high school	916 (46.4)	13.9		16.6	
Undergraduate or above	14 (0.7)	21.4		21.4	
Household income, 10,000RMB					
<3.0	340 (17.2)	12.1	<0.001	17.1	<0.001
3.0–7.0	807 (40.9)	17.1		25.4	
7.0–11.0	553 (28)	15.7		15.9	
≥11.0	273 (13.8)	6.2		4.8	
Smoking					
Never	1490 (75.5)	16.1	<0.001	20.3	
Sometimes	65 (3.3)	16.9		24.6	
Regular	418 (21.2)	7.7		10.8	
Alcohol drinking					
Never	1129 (57.2)	15.6		20.4	<0.001
Sometimes	514 (26.1)	18.7		22.8	
Regular	330 (16.7)	3.3		5.2	
Life satisfaction					
Very satisfied	1777 (90.1)	14.4	0.845	18.5	0.787
Basically satisfied	194 (9.8)	14.4		18.0	
Just so so	2 (0.1)	0.0			
Self‐rated health					
Good	1614 (81.8)	13.0	<0.001	14.7	<0.001
Just so so	359 (18.2)	20.3		35.1	

### 3.2 Anxiety and depression symptoms of the screening participants at baseline and follow‐up

**FIGURE 2:**
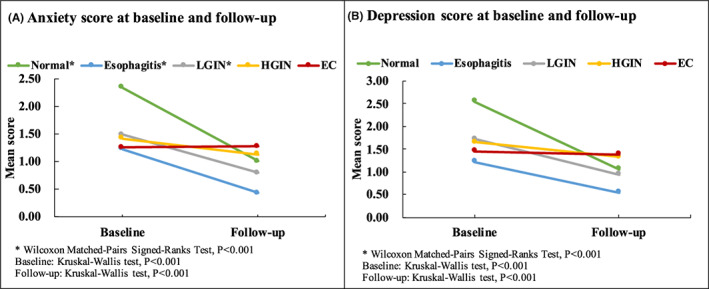
Anxiety and depression score at baseline and follow‐up, by esophageal pathology (A) Anxiety score at baseline and follow‐up; (B) Depression score at baseline and follow‐up

Table 2 compares the anxiety and depression symptoms of the screened participants at baseline and follow‐up. Compared to the results at baseline, it can be seen that the overall mean score and prevalence of anxiety decreased at follow‐up (1.67 [1.56–1.78] vs. 0.80 [0.72–0.88], *p* < 0.001; 14.3% [12.8%–15.9%] and 5.3% [4.3%–6.3%], *p* < 0.001). Similar results were found regarding depression symptoms. Further analysis by pathology grade showed that compared with baseline, the scores and prevalence of anxiety symptoms declined at follow‐up in participants screened as normal, participants with esophagitis, and participants with LGIN (normal: 2.34 [2.15–2.54] to 1.01 [0.84–1.19], 18.5%–7.1%; esophagitis: 1.23 [1.02–1.45] to 0.43 [0.29–0.56], 11.6%–2.1%; LGIN: 1.49 [1.30–1.69] to 0.80 [0.67–0.92], 13.1%–4.6%, all *p* < 0.001) (Table 2; Figure 2), but no significant difference in anxiety symptoms between baseline and follow‐up was observed for patients diagnosed with HGIN (1.42 [1.05–1.80] to 1.13 [0.78–1.48], *p* = 0.308; 13.5%–10.8%, *p* = 0.556) or EC (1.26 [0.61–1.92] to 1.28 [0.74–1.82], *p* = 0.710; 12.3%–8.8% *p* = 0.752). A similar trend was also found for depression symptoms.

**TABLE 2:** The anxiety and depression symptoms of the screening participants at baseline and follow‐up

	*N*	Baseline					Follow‐up					P3	P4
		Positive, *n*	Mean score (95% CI)	Prevalence (%, 95% CI)	P1	P2	Positive, *n*	Mean score (95% CI)	Prevalence (%, 95% CI)	P1	P2		
Anxiety symptoms													
All	1973	283	1.67 (1.56–1.78)	14.3 (12.8–15.9)			104	0.80 (0.72–0.88)	5.3 (4.3–6.3)			<0.001	<0.001
Normal	593	110	2.34 (2.15–2.54)	18.5 (15.4–21.7)	<0.001	0.012	42	1.01 (0.84–1.19)	7.1 (5.0–9.2)	<0.001	<0.001	<0.001	<0.001
Esophagitis	527	61	1.23 (1.02–1.45)	11.6 (8.8–14.3)			11	0.43 (0.29–0.56)	2.1 (0.9–3.3)			<0.001	<0.001
LGIN	649	85	1.49 (1.30–1.69)	13.1 (10.5–15.7)			30	0.80 (0.67–0.92)	4.6 (3.0–6.3)			<0.001	<0.001
HGIN	147	20	1.42 (1.05–1.80)	13.5 (7.9–19.1)			16	1.13 (0.78–1.48)	10.8 (5.7–15.9)			0.308	0.556
EC	57	7	1.26 (0.61–1.92)	12.3 (3.5–21.1)			5	1.28 (0.74–1.82)	8.8 (1.2–16.3)			0.710	0.752
Depression symptoms													
All	1973	364	1.82 (1.70–1.95)	18.4 (16.7–20.2)			107	0.91 (0.83–1.00)	5.4 (4.4–6.4)			<0.001	<0.001
Normal	593	150	2.55 (2.33–2.77)	25.3 (21.8–28.8)	<0.001	<0.001	36	1.06 (0.87–1.25)	6.1 (4.1–8.0)	<0.001	<0.001	<0.001	<0.001
Esophagitis	527	61	1.22 (1.01–1.43)	11.6 (8.8–14.3)			17	0.55 (0.39–0.70)	3.2 (1.7–4.7)			<0.001	<0.001
LGIN	649	116	1.72 (1.48–1.95)	17.9 (14.9–20.9)			29	0.94 (0.81–1.06)	4.5 (2.9–6.1)			<0.001	<0.001
HGIN	147	27	1.65 (1.21–2.10)	18.2 (11.9–24.5)			16	1.33 (0.99–1.68)	10.8 (5.7–15.9)			0.576	0.072
EC	57	10	1.46 (0.72–2.19)	17.5 (7.4–27.7)			9	1.39 (0.82–1.95)	15.8 (6.0–25.6)			0.230	1.000

*Note*: P1: Kruskal–Wallis test; P2: chi‐squared test; P3: Wilcoxon matched‐pairs signed‐ranks test; P4: McNemar's test.

Pairwise comparisons between the screening groups showed that the prevalence of anxiety and depression symptoms among the screening groups gradually distinguished at follow‐up. Significant differences in anxiety symptoms at follow‐up were observed in the following pairwise comparisons (all *p* < 0.001): normal versus esophagitis (7.1% vs 2.1%); esophagitis versus LGIN (2.1% vs 10.8%); esophagitis versus HGIN (2.1% vs 8.8%); LGIN versus HGIN (4.6% vs 10.8%). Significant differences in depression symptoms at follow‐up were observed in the following pairwise comparisons: esophagitis versus HGIN (3.2% vs 10.8%), esophagitis versus EC (3.2% vs 15.8%), LGIN versus HGIN (4.5% vs 10.8%), and LGIN versus EC (4.5% vs 15.8%) (Table 2).

### 3.3 Comparisons of the prevalence of anxiety and depression at follow‐up between the anxiety/depression group at baseline and the non‐anxiety/non‐depression group at baseline

In Table 3, we observed the prevalence of anxiety/ depression at follow‐up in the non‐anxiety/non‐depression group at baseline. The prevalence of anxiety at follow‐up in the non‐anxiety group was 4.7% (3.5%–5.7%). The corresponding prevalence of each pathology was 6.2% (4.1%–8.4%), 1.3% (0.3%–2.3%), 5.0% (3.2%–6.8%), 8.6% (3.7%–13.5%), and 8.0% (0.2%–15.8%) (*p* < 0.001); The prevalence of depression at follow‐up in non‐depression group was 4.8%. The corresponding prevalence of each pathology was 6.3%, 1.9%, 4.3%, 8.3%, and 14.9% (*p* < 0.001); The prevalence of anxiety or/and depression at follow‐up in non‐anxiety and non‐depression group was 6.9%. The corresponding prevalence of each pathology was 9.1%, 2.2%, 7.7%, 11.0%, and 15.2% (*p* < 0.001).

**TABLE 3:** Comparisons of the prevalence of anxiety/depression at follow‐up in non‐anxiety/non‐depression group at baseline

Baseline	Non‐anxiety group at baseline			Non‐depression group at baseline			Non‐anxiety and Non‐depression group at baseline		
Follow‐up	*n*	Prevalence of anxiety at follow‐up (%, 95% CI)	*p*	*n*	Prevalence of depression at follow‐up (%, 95% CI)	*p*	*n*	Prevalence of anxiety or/and depression at follow‐up (%, 95% CI)	*p*
Screeners	1690	4.7 (3.7–5.7)		1609	4.8 (3.7–5.8)		1541	6.9 (5.7–8.2)	
Esophageal pathological grade									
Normal	483	6.2 (4.1–8.4)	<0.001	443	6.3 (4.0–8.6)	<0.001	409	9.1 (6.3–11.8)	<0.001
Esophagitis	466	1.3 (0.3–2.3)		466	1.9 (0.7–3.2)		449	2.2 (0.9–3.6)	
LGIN	563	5.0 (3.2–6.8)		532	4.3 (2.6–6.1)		519	7.7 (5.4–10.0)	
HGIN	128	8.6 (3.7–13.5)		121	8.3 (3.3–13.2)		118	11.0 (5.3–16.7)	
EC	50	8.0 (0.2–15.8)		47	14.9 (4.3–25.5)		46	15.2 (4.4–26.0)	

In Table 4, we observed the prevalence of anxiety/ depression at follow‐up in anxiety/depression group at baseline. The prevalence of anxiety at follow‐up in anxiety group was 8.8% (5.5%–12.2%). The corresponding prevalence of each pathology was 10.9% (5.0%–16.8%), 8.2% (1.1%–15.3%), 2.4% (0.0%–5.6%), 25.0% (4.2%–45.8%), and 14.3% (0.0%–49.2%) (*p* = 0.019); The prevalence of depression at follow‐up in depression group was 8.2%. The corresponding prevalence of each pathology was 5.3%, 13.1%, 5.2%, 22.2%, and 20.0% (*p* = 0.008); The prevalence of anxiety or/and depression at follow‐up in anxiety or/and depression group was 11.8%. The corresponding prevalence of each pathology was 10.3%, 12.8%, 6.9%, 31.0%, and 36.4% (*p* < 0.001).

**TABLE 4:** Comparisons of the prevalence of anxiety/depression at follow‐up in anxiety/depression group at baseline^a^

Baseline	Anxiety group at baseline			Depression group at baseline			Anxiety or/and depression group at baseline		
Follow‐up	*n*	Prevalence of anxiety at follow‐up (%, 95%CI)	*p*	*n*	Prevalence of depression at follow‐up (%, 95%CI)	*p*	*n*	Prevalence of anxiety or/and depression at follow‐up (%, 95%CI)	*p*
Screeners	283	8.8 (5.5–12.2)		364	8.2 (5.4–11.1)		432	11.8 (8.8–14.9)	
Esophageal pathological grade									
Normal	110	10.9 (5.0–16.8)	0.019	150	5.3 (1.7–9.0)	0.008	184	10.3 (5.9–14.8)	<0.001
Esophagitis	61	8.2 (1.1–15.3)		61	13.1 (4.4–21.8)		78	12.8 (5.2–20.4)	
LGIN	85	2.4 (0.0–5.6)		116	5.2 (1.1–9.3)		130	6.9 (2.5–11.3)	
HGIN	20	25.0 (4.2–45.8)		27	22.2 (5.5–39.0)		29	31.0 (13.1–48.9)	
EC	7	14.3 (0.0–49.2)		10	20.0 (0.0–50.2)		11	36.4 (2.5–70.3)	

^a^ The prevalence of anxiety/depression at follow‐up is higher in anxiety/depression groups at baseline than that in non‐anxiety/non‐depression group at baseline (8.8% vs 4.7%, *p* = 0.004; 8.2% vs 4.8%, *p* = 0.009).

The prevalence of anxiety at follow‐up was higher in the groups of patients who had anxiety at baseline than in the groups of patients without anxiety at baseline (8.8% vs 4.7%, *p* = 0.004). Similarly, the depression group at baseline had a higher prevalence of depression symptoms at follow‐up than the non‐depression group (8.2% vs 4.8%, *p* = 0.009). In addition, the prevalence of anxiety and depression symptoms at follow‐up was different among participants with various pathology grades (all *p* < 0.05). The prevalence of anxiety at follow‐up in the anxiety group at baseline and the non‐anxiety group at baseline were 10.9% and 6.2% for normal (*p* = 0.083), 8.2% and 1.3% for esophagitis (*p* < 0.001), 2.4% and 5.0% for LGIN (*p* = 0.285), 25.0% and 8.6% for HGIN (*p* = 0.029) and 14.3% and 8.0% for EC (*p* = 0.494). Similar patterns were found for depression symptoms.

**FIGURE 3:**
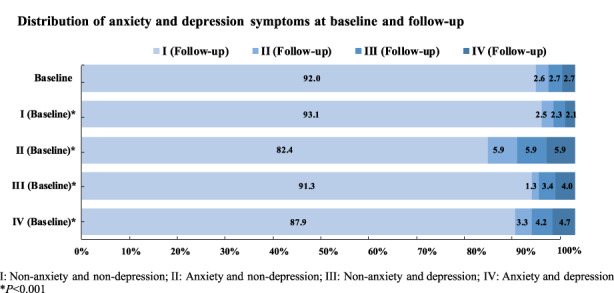
Distribution and variation of anxiety and depression symptoms at baseline and follow‐up

Considering the overlap of anxiety and depression symptoms, we also divided the participants into four groups (I–IV) based on anxiety and depression symptoms in Figure 3: non‐anxiety and non‐depression (I), anxiety and non‐depression (II), depression and non‐anxiety (III), and anxiety and depression (IV). There was a significant difference in the distribution of anxiety and depression at follow‐up in different anxiety and depression groups at baseline (*p* < 0.001). The majority of patients in groups I/II/III/IV at baseline transitioned to Group I at follow‐up.

### 3.4 The risk of anxiety and depression symptoms due to endoscopic screening and disease diagnosis

As shown in Table 5, after adjusting for possible confounding factors, a comparison of the screened population at baseline and follow‐up showed that the overall psychological impact of screening weakened for anxiety (RR = 0.37 [0.30–0.46], *p* < 0.001) and depression (0.29 [0.24–0.36], *p* < 0.001) during follow‐up. However, compared with baseline psychological symptoms, the high‐level anxiety and depression symptoms remained at follow‐up for patients diagnosed with HGIN (anxiety: 0.80 [0.13–1.48], *p* = 0.477; depression: 0.59 [0.33–1.05], *p* = 0.070) and those diagnosed with EC (anxiety: 0.71 [0.24–2.12], *p* = 0.542; depression: 0.90 [0.40–2.05], *p* = 0.802).

**TABLE 5:**
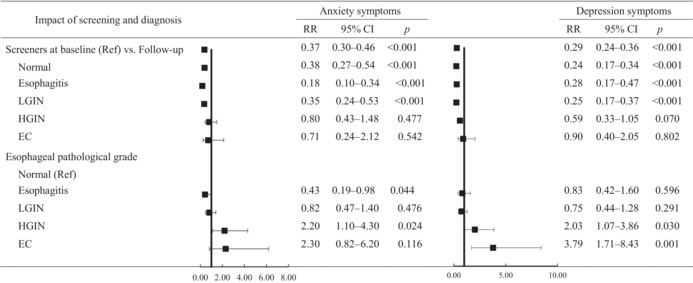
The risk for anxiety and depression symptoms due to endoscopic screening and diagnosis

*Note*: Region, age, gender, household income, smoking, alcohol drinking, and self‐rated health were adjusted.

Taking screening tools diagnosed as normal as a reference, we found that the risk of anxiety and depression increased with pathology grade, although there may be no significant difference. Compared with participants classified as normal, for patients with esophagitis, LGIN, HGIN, and EC, the RRs of anxiety for each pathology grade were 0.43, 0.82, 2.20, and 2.30, respectively, and the corresponding RRs of depression symptoms for each pathology grade were 0.83, 0.75, 2.03, and 3.79, respectively.

## 4 DISCUSSION

This multicenter, population‐based study showed that there was a downward trend in anxiety and depression symptoms for participants of EC screening over time, except in patients with HGIN and patients with EC (for whom the anxiety and depression symptoms remained high). The findings fill a gap in this field. The timely assessment of psychological concerns and implementation of interventions are of great importance in the long run for individuals who are ready for screening and those diagnosed as having HGIN or EC. Our findings provide a scientific basis and vital clues for the optimization of upper gastrointestinal cancer screening.

A contradictory study that reviewed psychological distress related to cancer screening indicated that psychological distress was low across screening procedures.^11^ Possible explanations for the contradictory findings were that many unvalidated measures were used in the review's eligible studies. In addition, EC screening was not included in the systematic review. EC and colorectal cancer have similarities in their screening techniques (both involve invasive endoscopy). Clearly, an invasive examination easily causes people to be nervous and distressed. They are afraid of bad screening results, which might even make them give up halfway.^12^ Similar results have been found in patients ready for PET and CT examinations; they have increased anxiety.^18^ Their over‐anxiousness before the screen may only be partly due to the endoscopic process, but mainly due to their fear of getting cancer. Such influence would be more severe among clinical samples, because the over‐anxiousness before the screen was due to their baseline anxiety over diseases or other possible confounders not included in the current study, and not necessarily due to the anxiety towards the endoscopic screen. Therefore, we would improve the survey on the psychological impact of screening, and add survey variables such as the fear level of invasive endoscopy and the degree of worry about screening results. Several plausible biological mechanisms have been proposed regarding this association.^19^, ^20^, ^21^, ^22^, ^23^ A cancer‐related diagnosis at screening may act as a serious stressor and stimulate stressful life events, especially for patients screened as having HGIN or EC. A potential direct effect on neuroendocrine‐immune processes, such as the hypothalamic–pituitary–adrenal axis (HPA)and exorbitant levels of cortisol. Anxiety and cancer may both result from stress that triggers neuroendocrine processes.^19^, ^20^, ^21^, ^22^, ^23^

The invasive procedure is a major concern for the low participation rate of endoscopic screening.^12^, ^24^ The preoperative psychological emotions (increased anxiety and depression) caused by invasive endoscopic screening largely play a role in the low participation rate and compliance.^12^, ^24^ Several large‐scale population‐based studies indicated that only 33.5% and 18.4% of participants were eventually screened by endoscopy.^5^, ^25^ The participation rate for nationwide endoscopic screening in Korea was still low (11.4% in 2002; 29.2% in 2008; 47.3% in 2012).^7^, ^26^ In Japan, endoscopy has been partially adopted in population‐based screening, with a participation rate of 16%.^27^ Similar results were identified in colorectal cancer screening in China (14.0%),^28^ which can be in line with the rate for endoscopic screening of other countries above. Low participation wastes resources and has an inconspicuous influence on the reduction in mortality.^5^ In the study, we found anxiety and depression weakened over time among healthy people and participants with esophagitis, which suggested endoscopic screening might actually help ease the anxiety and depression in those that received the screening and found themselves without HGIN or EC despite the fear for the invasiveness of the screening. Besides, a non‐invasive, simple, effective, and novel screening technique should be developed and updated to improve compliance in the future. For example, in recent years, the emerging magnetic control capsule endoscopy, which has high acceptance and compliance, has demonstrated good efficiency and accuracy in multicenter field verification in China.^29^

Anxiety and depression may have a negative impact on sleep, daily life, and both physical and mental health.^30^, ^31^ Anxiety and depression may play vital and unfavorable roles in increasing the risk of cancer incidence, the receipt of subsequent treatments.^32^, ^33^, ^34^, ^35^, ^36^ For screened patients with less serious conditions, their moods could recover at follow‐up, but the psychological symptoms of patients with higher grade (HGIN or EC) pathologies were not alleviated over time, which has adverse effects on prognosis and survival. One possible explanation is that the HGIN and EC diagnosis was a stressful life event and stimulate their nerves, increasing their anxiety and depression.^21^, ^22^, ^23^ A pooling analysis from 16 prospective cohorts provided evidence in support of our view and addressed the key role of anxiety and depression symptoms as a potential predictor of EC mortality (HR = 2.59, 1.34–5.00).^37^ In addition, studies have shown that reducing anxiety or depression can bring benefits to patients. Therefore, it is necessary to improve the emotional health of screened individuals. Sufficient psychological surveillance and timely care should be given priority in vulnerable groups, including those who are ready for screening and those screened as having HGIN or EC; for example, smart bracelet monitoring may help relieve their psychological burden.

Health education and psychological interventions may be good choices for improving psychological distress. In high‐incidence areas of China, residents lack accurate and fundamental knowledge related to esophageal precancerous lesions and cancer, and their levels of health awareness and literacy are low. The “cancer fear” phenomenon is very common. The compliance with EC screening was largely depended on participants' knowledge and awareness on cancer prevention.^38^ Therefore, publicity campaigns that promoted cancer prevention seemed to be beneficial to the optimization of EC screening.^39^ It is highly advisable for policy‐makers to pay more attention to this important issue.

In this study, we found differences in anxiety and depression symptoms at baseline among the various regions. The possible reasons are as follows: (a) The prevalence of EC and the detection of lesions were different in the various regions. Among the five sites, the prevalence of EC was the highest in Linzhou, which may have caused the levels of anxiety and depression symptoms in patients in Linzhou to be the most serious. Linzhou, as a representative high‐incidence region, is the most typical area with a high incidence of EC in China. Therefore, EC may place a heavier psychological burden on local residents. They suffered and feared the high risk of EC. (b) Considering the differences in geography, personnel ability and healthcare facilities, these factors may lead to differences in residents' anxiety and depression symptoms and an imbalance in the sample collection among the sites. For example, Yanting is located in Sichuan in western China, which is a mountainous area. Advanced medical resources were limited, which may affect the residents' psychological burden. There are many influencing factors related to the prevalence of anxiety and depression symptoms that vary among the different regions. The results should be interpreted carefully.

A strength of this study is that, to our knowledge, it is the first population‐based, multicenter prospective cohort investigation conducted to evaluate the psychological effects of endoscopic screening for EC on anxiety and depression symptoms. Second, based on its endoscopic screening protocol in multiple areas of China with a high incidence of EC, the design is innovative and convincing to some extent. For consistency and accuracy, measures were evaluated before the actual screening procedure. The third advantage is the use of a comparative approach to evaluate the impact of endoscopic screening in various screening groups. Fourth, both anxiety and depression symptoms, which are major manifestations of anxiety and depression symptoms, were all measured by validated instruments, and multiple measurements were made in this study. The GAD‐7 and PHQ‐9 have emerged as powerful tools for studying anxiety and depression disorders. In addition, the consistent results of anxiety and depression symptoms make the results more credible.

Some limitations exist in the study. First, a major problem is selection bias and volunteer bias. Causal inferences cannot be determined. Second, comparisons between screened participants and controls were confined in the study. We collected a small number of controls in the pre‐experiment phase and will expand the sample size in the future. Third, GAD‐7 and PHQ‐9 are just screening instruments for symptoms of anxiety or depression, and not clinical disorders. The quantitative investigation combined with a clinical interview by psychiatrists could be considered in the future screening process. Fourth, the cut‐off point of five may overestimate the results of anxiety and depression symptoms. Finally, only once follow‐up in the study, multi‐stage follow‐up may be convincing (6 months, 12 months, 18 months, etc.). Considering the limitations on the current design, the results may need to be interpreted with caution. Large controls who did not participate in the screening were needed. Unmeasured and residual confounding may interfere with the interpretation and validity of the results. Biological mechanisms need further exploration in the future.

In conclusion, there was a downward trend in these detrimental effects over time. Of particular concern are individuals who are ready for screening and those screened as having HGIN or EC. The study offered a fresh perspective on EC screening and provided new insights into optimizing endoscopic screening procedures. Suggestions were identified to remedy these problems in future studies. Feasible psychological interventions and attempts to encourage endoscopic screening are urgently needed. The study provides practical evidence for the optimization of upper gastrointestinal screening and the minimization of psychological harm, and it provides a reference for other developing countries with similar patterns of upper gastrointestinal cancers.

## AUTHOR CONTRIBUTIONS

**Juan Zhu:** Conceptualization (lead); data curation (lead); formal analysis (lead); funding acquisition (supporting); investigation (lead); methodology (lead); project administration (equal); supervision (equal); visualization (equal); writing – original draft (equal); writing – review and editing (equal). **Shanrui Ma:** Project administration (equal); supervision (equal). **Ru Chen:** Project administration (equal); resources (equal); supervision (equal). **Shuanghua Xie:** Project administration (equal); resources (equal); supervision (equal). **Zhengkui Liu:** Conceptualization (equal); methodology (equal); writing – review and editing (equal). **Zhaorui Liu:** Conceptualization (equal); methodology (equal); writing – review and editing (equal). **Wenqiang Wei:** Conceptualization (equal); formal analysis (equal); funding acquisition (lead); investigation (equal); methodology (equal); project administration (lead); resources (equal); supervision (equal); validation (equal); visualization (equal); writing – original draft (equal); writing – review and editing (equal).

## FUNDING INFORMATION

This study was supported by the National Key Research and Development Program (Precision Medicine Research), Grant/Award Number: 2016YFC0901400, 2016YFC0901404.

## CONFLICT OF INTEREST

The authors declare no potential conflict of interest.

## ETHICAL STATEMENT

The study was approved by the National Cancer Center/National Clinical Research Center for Cancer/Cancer Hospital, Chinese Academy of Medical Sciences and Peking Union Medical College (no. 16‐171/1250). All procedures involving human participants were performed in accordance with the ethical committee's standards and the Helsinki declaration. All participants signed informed consent.

## Data Availability

Source data are available from the corresponding author on reasonable request.
